# Making a HIIT: co-design of high-intensity interval training workouts with students & teachers within the curriculum

**DOI:** 10.1186/s12889-023-16613-8

**Published:** 2023-09-15

**Authors:** Stephanie L. Duncombe, Alan R. Barker, Lisa Price, Jacqueline L. Walker, Yong Liu, Dewi Paris, Michalis Stylianou

**Affiliations:** 1 School of Human Movement and Nutrition Sciences, University of Queensland, Saint Lucia, QLD 4072 Australia; 2 Children’s Health and Exercise Research Centre, Public Health and Sport Sciences, University of Exeter, Exeter, UK

**Keywords:** Youth, School, Feasibility, Physical education, Physical activity

## Abstract

**Background:**

High-intensity interval training (HIIT) interventions are becoming more common in schools. However, limited input has been sought from end-users, which can help design interventions that are more engaging and context appropriate, therefore increasing their potential for successful implementation. One method of engaging end-users is co-design, which involves an active collaboration to design solutions to pre-specified problems. This paper aimed to: (1) describe the methodology and results of the co-design process in *Making a HIIT* to develop HIIT workouts for a school-based intervention; and (2) evaluate the feasibility and impact of co-designing HIIT workouts with students and teachers within the health and physical education (HPE) curriculum.

**Methods:**

The development of the HIIT workouts occurred during obligatory HPE lessons with year seven and eight students. The co-design process included: (1) identifying barriers and facilitators to exercise to create evaluation criteria for creating the HIIT workouts; (2) exploring HIIT; (3) defining HIIT parameters (intensity and interval length); (4) creating HIIT workouts using the parameters and evaluation criteria; (5) trialling and modifying the HIIT workouts based on class feedback and intensity data. To evaluate the feasibility and impact of the co-design process, a thematic analysis was completed using teacher interviews, student discussions, and student surveys.

**Results:**

Five classes comprised of 121 students (12–14 years; 49% female) and five teachers were involved in the co-design process across three schools in Queensland, Australia. A total of 33 HIIT workouts were created aimed at satisfying the HIIT parameters and variations of the following evaluation criteria: (1) fun; (2) social; (3) achievable skill level; (4) feeling accomplished; and (5) beneficial for health. From the thematic analysis, three themes (acceptability; implementation; integration) and 12 codes contributed to the overarching understanding of the feasibility of the lessons within the curriculum and a further three themes (perceived changes to lessons; educative outcomes; personal and social capabilities) and three codes contributed towards understanding their impact.

**Conclusion:**

Overall, co-designing HIIT workouts was feasible within the HPE curriculum and may have contributed to positive educative outcomes. Using this methodology could improve the implementation of HIIT interventions within HPE while supporting educative benefits.

**Supplementary Information:**

The online version contains supplementary material available at 10.1186/s12889-023-16613-8.

## Background

Most children and adolescents are not acquiring the amount of moderate-to-vigorous physical activity recommended for health benefits [1–3]. Recent evidence has demonstrated that vigorous physical activity specifically could be driving some of the health benefits, such as improved cardiorespiratory fitness and body composition [4, 5], suggesting that developing interventions that focus on promoting vigorous physical activity are necessary. High-intensity interval training (HIIT) is a form of vigorous physical activity that incorporates alternating bouts of high-intensity exercise and recovery [6]. HIIT follows a similar intermittent pattern to children’s habitual physical activity and has been used in school-based interventions [7, 8].

Schools are an important setting for acquiring physical activity. They can reach a high percentage of children and adolescents with their policies, infrastructure, and trainable personnel [9]. However, the school environment also presents unique challenges, including time constraints, teacher workload, and curriculum demands [10]. Currently, most school-based HIIT interventions have demonstrated limited consideration of these challenges and have had minimal input from students and teachers to tailor the interventions for student enjoyment or to curriculum units [8]. Of the 42 studies identified in a systematic review on school-based HIIT [8], only two had included any engagement with end-users during design and implementation [11, 12]. Therefore, contemporary reviews have recommended integrating HIIT within the curriculum and consulting teachers and students [8, 13, 14]. The Australian Health and Physical Education (HPE) curriculum includes standards and elements related to fitness and the benefits of physical activity for health [15], which presents an opportunity for the integration of HIIT and active involvement of students and teachers while still focusing on educative outcomes.

Participation with end-users, such as teachers and students, occurs on a 5-stage continuum outlined by the International Association for Public Participation (IAP2), which includes informing, consulting, involving, collaborating, and empowering [16]. While integrating interventions within the curriculum has the potential to alleviate some of the challenges outlined above, time restraints and assessment requirements restrict student involvement, limiting the feasibility of stage 5 (empowering) [17]. Young people have previously been included in the development of school-based physical activity interventions; however, this rarely occurs beyond stage 2 (consultation) [18]. Including students and teachers at higher levels on the IAP2 continuum can provide several benefits, including: (1) providing students with a voice to express their needs [19]; (2) increasing students’ confidence [19]; (3) increasing skill acquisition for students [19]; and (4) enhancing relevant projects through a better understanding of student and teacher needs by involving them as experts [17]. However, it is important to ensure that the involvement of end-users is authentic, which can be fostered through practical strategies and frameworks that guide the process [20, 21].

The *Making a HIIT* study described in this paper was designed to enable authentic end-user participation through co-design. Co-design is defined as a collective creativity across the entire design process and involves an active collaboration with end-users to design solutions to pre-specified problems [22, 23]. It is distinguished from other forms of end-user engagement such as co-creation and co-production that have differing levels of end-user participation. Co-creation engages end-users before the problem is identified and necessitates the highest level of engagement from end-users [23]. Conversely, co-production requires less engagement from end-users and involves them in the evaluation of potential solutions to a problem [23]. In this instance, co-design was deemed to enable sufficient and meaningful participation from end-users, while complementing other curriculum demands. For the *Making a HIIT* study, it enabled the expertise and lived experiences of researchers, teachers, and students to be combined to create and use HIIT workouts within the HPE curriculum. If done appropriately, the co-design process has the potential to lead to the development of HIIT workouts that are more engaging and useful to students and teachers by bringing together different views, contributions, and expertise [24].

This paper presents the co-design approach from the *Making a HIIT* study where HIIT workouts were co-designed within the HPE curriculum by students, teachers, and researchers. This paper aims to: (1) describe the methodology and results of the co-design process; and (2) evaluate the feasibility and impact of co-designing HIIT workouts with students and teachers as part of the HPE curriculum.

## Methods

The overall study design of *Making a HIIT* has previously been described [25]. This paper focuses on phase one of the study. This phase was completed within obligatory HPE lessons and involved co-designing HIIT workouts with students and teachers within the curriculum, which were subsequently used in an intervention in phase two of *Making a HIIT*. A brief overview of the topics covered in the phase one lessons is presented in Table 1.

**Table 1:** Topics covered in the high-intensity interval training co-design lessons

Topic	Key actions
**Problem Identification** Theory lesson	• The co-design team listed their three main barriers and facilitators to exercise on individual sticky notes. • The co-design team visually grouped their barriers and facilitators into themes to understand which ideas were the most common. • The co-design team used the common barriers and facilitators to collectively create workout criteria that represented their shared thoughts.
**Upskilling** Practical lesson	• Students used heart rate monitors to familiarise themselves with heart rate and high-intensity and experiment with achieving different heart rate zones. • The co-design team partook in several HIIT workouts, reflected on their heart rate, and rated the workouts using the criteria developed during problem identification.
**HIIT Design** Theory and practical lesson	• The co-design team collectively decided on the percentage of heart rate maximum that was classified as high-intensity, and the minimum and maximum interval lengths that could be used in their workouts. • In small groups, students identified potential themes for their HIIT workout and exercises that fit the theme. • Using their identified themes and exercises, the small groups created a HIIT workout that was consistent with the established HIIT parameters and attempted to satisfy the criteria developed during problem identification.
**HIIT Piloting** Practical lesson	• Each small group led their HIIT workout for the co-design team with the aid of the teacher and researcher. • The co-design team provided feedback on other groups’ HIIT workouts using the criteria developed during problem identification. • Heart rate for each pilot was recorded.
**HIIT Modification** Theory lesson	• All groups modified their workouts based on: o their own opinion of their pilot. o the feedback provided by the co-design team during the pilot. o the heart rate summary data from their pilot.

*HIIT *High-intensity interval training

The co-design process was guided by the framework and recommendations from Leask et al. [21] and adapted at each school to meet their specific needs. This framework was designed to systematically guide the development of public health interventions using participatory methods and was informed by several case studies, including one focused on physical activity in secondary schools with students as the end users [21]. While this framework has a focus on participatory action research, which aims to change social reality by means of participatory research [26], it includes four main stages, which are all relevant to co-design: (1) planning; (2) conducting; (3) reporting; and (4) evaluating. These stages are presented in Table 2, where they are linked to the corresponding information of the co-design process for *Making a HIIT*.

**Table 2:** Operationalisation of the Leask et al. framework for the co-design of high-intensity interval training workouts

			HIIT Workout Co-Design
Planning	Aim of Study	Problem	A low percentage of students are meeting the recommend minutes of moderate-to-vigorous physical activity to acquire health benefits
		Objective	To co-design high intensity interval training (HIIT) workouts in health and physical education (HPE) lessons that can be used in HPE lessons
		Design	Co-design
		End Users	HPE teachers and students
		Co-designers	Researchers, HPE teachers, students
		Evaluation	The process was evaluated with individual student surveys, student discussions, and teacher interviews
		Scalability	Two outputs from this study have the potential to be scaled for further school-based HIIT interventions: 1. The co-design process and the associated lessons plans that can be integrated in the HPE curriculum across multiple schools, which were refined throughout the three consecutive schools in *Making a HIIT* 2. The HIIT workouts designed by the co-design team that focused on incorporating facilitators to exercise and alleviating barriers can be shared between teachers to be used as brain breaks, warmups, or in HPE
	Recruit/ Sampling Procedure	Criteria	All students within the co-design classes at participating schools were eligible to participate
		Setting	HPE lessons in three greater Brisbane schools
		No. of creators	A researcher, one class of Year 7 or 8 students, and the class teacher formed an independent co-design team
		Demographics/characteristics	Broad and generalisable schools: a co-educational state school, a girls’ catholic school, and a boys’ independent school with different levels of socio-educational advantage (Table 3)
Conducting	Ownership	Manifesting Ownership	Ownership was established using several methods: 1. The HIIT parameters were decided using participation from all team members with each team member receiving a vote. 2. Depending on the class, students were given the opportunity to decide whether to form groups themselves or have their groups formed by a teacher 3. Groups decided on a team name and theme collectively that reflected shared interests and the personality of the group 4. Groups had the final decision on the theme and exercises included in their workouts based on input from the rest of the co-design team 5. Index cards at the end of the lessons were provided for anonymous student feedback that could be discussed the following session
	Procedural Components	Level of participation	Team-based design with the researcher as facilitator and teacher as facilitator/collaborator with all members of the team, including students, treated as experts
		How was the aim presented?	The objective for the co-design process was discussed with all co-designers at the start of the first session along with the proposed lesson plans. The co-design team (students and teachers) was asked to provide feedback on the aims and activities included the lessons.
		Purpose of each meeting presented	Each lesson commenced with a “what, why, how” discussion to ensure that it had a clear purpose, rationale, and outline of activities
		Rules and responsibilities agreed upon?	All co-designers were of equal status and had the right to contribute ideas. This was actioned by: 1. Ensuring each team member had a vote for the creation of the criteria and HIIT parameters 2. Ensuring each team member in the HIIT creation groups had an agreed upon role and provided ideas for included exercises
	Procedural Methods	Upskilling	Student co-designers deepened their understanding of HIIT by: 1. Increasing their understanding of heart rate intensity zones using heart rate monitors 2. Discussing (1) high intensity; (2) intervals; and (3) the relationship between rest, work, and intensity 3. Trialling a range of workouts that fall under the definition of HIIT
		How was previous evidence reviewed?	1. A systematic review and meta-analysis of school-based HIIT was completed by researchers that identified a lack of student and teacher voice and integration within the curriculum [8] 2. Lived experiences around barriers and facilitators to exercise were discussed with the co-design team and the most common barriers and facilitators were identified visually with sticky notes
		Prototype Process	To design the HIIT workouts at a single school: 1. Co-design teams made their 1st iteration of the HIIT workouts 2. The HIIT workouts were piloted with the co-design team 3. Each group received the heart rate data from their HIIT workout and feedback from the co-design team based on the class criteria 4. The teams modified their HIIT workouts based on the feedback and heart rate data To improve the co-design process: 1. The co-design process was conducted at a single school 2. Teachers and students provided feedback on the process 3. The process was modified prior to being implemented at the next school
		Frequency and duration of lessons	The lessons varied between schools based on decisions with teachers and head of department. They were: 1. School 1: 6 × 70-minute lessons 2. School 2: 6 × 50-minute lessons 3. School 3: 6 × 60-minute lessons
		Interactive techniques used	Active participation in activities both in theory (standing on a line to indicate thoughts about intensity and discussing with peers; physically grouping facilitators and barriers to exercise using sticky notes) and practical lessons (leading workouts; visual representation of heart rate zones)
		Fieldwork techniques used	1. Heart rate exploration was completed in the upskilling lesson 2. Pre-made HIIT workouts were trialled in the upskilling lesson 3. Groups piloted their HIIT workouts with their peers, teacher, and researcher
		How did iteration occur?	1. Criteria for creating the HIIT workouts were developed, piloted, and revised/finalised 2. HIIT workouts were developed, piloted, and revised/finalised
Evaluation	Process Evaluation	Co-design process evaluated?	Evaluation of the feasibility and impact of the co-design process was completed using qualitative analysis. Data included: 1. Written surveys completed by each participating student 2. Notes from the student discussions with researchers in their small co-design groups 3. Audio recordings of semi-structured interviews with co-design teachers The co-design process was implemented at each school consecutively with feedback from one school used at the following
		Results reported to stakeholders/public?	1. The final HIIT workouts were shared with the teachers involved in the co-design process and the head of the HPE department to distribute and use as they preferred. 2. The feasibility and impact of the process was discussed with stakeholders (teachers) and the findings of the discussions were relayed to other stakeholders (e.g., the head of the department) 3. Dissemination of the methods and findings of the study will be completed via journal articles and conference presentations.
	Outcome Evaluation	Validity of outcome (HIIT Workouts)	1. The HIIT workouts included multiple iterations and modifications to increase the likelihood that they satisfied the HIIT parameters and class evaluation criteria 2. A second phased of *Making a HIIT* will embed the co-designed HIIT workouts in an experimental study and evaluate fidelity and workout quality
		Plan to test effectiveness/scalability of outcome?	1. A second phase of this study will embed the co-designed HIIT workouts in an experimental study comparing the motivation and enjoyment of co-designers to students not involved in co-design 2. A second phase of this study will embed the co-designed HIIT workouts in an experimental study comparing the fitness and executive function of students completing the co-designed HIIT sessions to a control group

The recommended framework outlined by Leask et al. [21] and the corresponding methods and activities completed in this study. *HIIT *High-intensity interval training, *HPE *Health and physical education

## Recruitment and sampling procedure

Three metropolitan secondary schools in Queensland, Australia were recruited through purposeful sampling to participate in *Making a HIIT* and their characteristics are displayed in Table 3. The classes that participated in the co-design process were chosen in consultation with teachers based on lesson schedules. Each class constituted its own co-design team that included all the students, the teacher, and the researcher. Ethical approval for the study was granted by The University of Queensland’s human research ethics committee (Project: 2020/HE002444) and relevant education organisations. All students and teachers in the partaking classes were eligible to participate. Informed consent was collected from teachers and school principals. Informed assent and consent were obtained from students and their parents / guardians. In total, 121 of a possible 129 students, and five teachers were involved in the co-design process across the three schools. No students or teachers withdrew from the process.

**Table 3:** School and class characteristics

School	School Type	ICSEA percentile^a^	Language Other than English^a^	Class	Year Level (Mean age)	*N* (girls)	Agreed Upon HIIT Parameters		
							Intensity Threshold	Min Interval Length	Max Interval Length
One	State	41%	42%	A	8 (13.3 ± 0.3)	25 (11)	80% of HR_max_	10 s	60 s
Two	Independent	87%	24%	B	7 (12.6 ± 0.3)	24 (0)	85% of HR_max_	10 s	60 s
				C	7 (12.5 ± 0.3)	24 (0)	90% of HR_max_	10 s	60 s
Three	Catholic Education	65%	5%	D	8 (13.3 ± 0.3)	23 (23)	80% of HR_max_	10 s	60 s
				E	8 (13.4 ± 0.3)	25 (25)	80% of HR_max_	10 s	60 s

The values presented in the school information columns were acquired from myschool.edu.au. The HIIT parameters for each class were decided by each co-design team. *ICSEA *Index of Community Socio-Educational Advantage, *SEA *Socio-educational advantage, *HIIT *High-intensity interval training, *HR *Heart rate, *Max *Maximumm *Min *Minimum, *N *Number of students

^a^ Based on 2021/2022 results from: https://myschool.edu.au/

### Procedural components

While a similar lesson structure was observed across participating classes, a separate co-design process was conducted for each one with no influence from the other classes on the decisions made. The lesson plans were developed by the research team in consultation with the head of the HPE department and participating HPE teachers at each school, and in alignment with content descriptors from the Australian HPE curriculum for Years 7 and 8 (e.g., designing personal fitness plans and modifying systems to enable enjoyment and success) [15]. The lesson plans designed, including the activities and resources, are available from the research team upon reasonable request. The number of lessons for the co-design process was determined by the researchers and teachers prior to interacting with students as it needed to be decided before the start of the term so teachers could plan the remaining lessons and their assessments. At the beginning of the co-design procedure, the co-design team discussed the objectives and tentative lesson plans as outlined in Table 1. Students were encouraged to provide their thoughts and feedback on the lessons throughout the co-design process, either through class discussions or anonymously using index cards that could be discussed during the next lesson. At the start of each lesson, the “what, why, and how” were discussed so that all members of the co-design team were clear on the purpose of the lesson, underlying rationale for the lesson, and associated activities. Researchers facilitated the lessons with support from the HPE teacher, who held a passive role throughout the lessons to minimise the influence of teacher-student power dynamics. Students’ lived experiences and input were treated as equally important to that of researchers’ and teachers’, and as essential to co-designing the workouts.

### Procedural methods

#### Frequency and duration of lessons

The intention was to conduct the co-design process across 6 HPE lessons as part of the curriculum. In school one, the co-design team met twice a week for three weeks and each HPE lesson was 70 min. In school two, one class completed six 50-minute lessons and one class completed only 5 lessons due to scheduling conflicts disrupting class time. The lessons in school two were completed across five weeks and occurred between one to three times per week. In school three, both classes met twice a week for three weeks and each HPE lesson was 60 min. In all three schools, the process was integrated within a fitness-related unit and was completed in both theory and practical lessons using a classroom and the school gymnasium.

#### Problem identification: HIIT criteria creation

Prior to beginning this study, the research team completed a systematic review of the school-based HIIT literature and identified a lack of student and teacher voice and integration within the curriculum [8]. With the purpose of creating workouts centred on student interests and enjoyment, the first lesson of the co-design process started with a focus on barriers and facilitators to general exercise to create criteria for engaging exercise workouts as described in Table 1. Students used the created criteria to evaluate several pre-made HIIT workouts during the upskilling lesson. Subsequently, the class discussed what modifications, if any, were needed to properly represent their interests before the criteria were used to inform the design of their own HIIT workouts. Data collected on this topic included the sticky notes listing each student’s individual barriers and facilitators, the draft class criteria, and the final class criteria.

#### HIIT upskilling

Prior to designing the HIIT workouts, the second lesson was used to familiarise students in the co-design team with heart rate and HIIT. The co-design team discussed resting heart rate and calculated their estimated maximum heart rate in beats per minute. Each student was provided with a Polar H10 heart rate monitor (Polar H10, Polar Electro, Finland) and instructed on proper placement. Using Polar GoFit software (https://polargofit.com/), students heart rates were anonymously projected in the gymnasium (i.e., using assigned numbers instead of names). The GoFit software uses different coloured zones to denote 90%+, 80 − 89%, 70–79%, 60–69%, and < 60% heart rate, so students could quickly determine their effort while working and resting. Students had a chance to freely move around the gymnasium and watch their heart rate response on screen with the goal of displaying all five heart rate zones.

To understand HIIT, the researchers then introduced the students in the co-design team to the concept of intervals and the co-design team discussed the relationship between intensity and interval length. The co-design team discussed how heart rate and intervals were relevant to HIIT and co-design team members identified any prior knowledge or experiences they had of HIIT from gyms, social media, or other sources. Students in the co-design team were never provided with a specific definition of HIIT (e.g., an intensity threshold) from the researcher so that they would be able to formulate their own definition based on their knowledge from this lesson. Students in the co-design team did trial a variety of HIIT workouts chosen by the research team to gain a greater understanding of the types of exercises in HIIT workouts and how they influenced heart rate. In school one this included a: (1) relay for points; (2) resistance workout; (3) dance-themed workout; (4) boxing themed workout; and (5) run/jog workout. Due to time constraints, the boxing and dance workouts were not completed in school two, and the dance workout was not completed in school three. Students used the criteria created in the first lesson to evaluate the workouts. The data collected in this lesson included the evaluation page that each student completed for each HIIT workout using the criteria created by each class.

#### HIIT workout parameters

During the third lesson, the co-design team collectively decided on: (1) the threshold for high intensity as a percentage of maximum heart rate; (2) the maximum interval length for work and rest; and (3) the minimum interval length for work and rest. Using their understanding of heart rate from the HIIT workouts in the upskilling lesson, students lined up on a continuum across the classroom to mark where they thought the threshold for high intensity should be set as a percentage of maximum heart rate ranging from 50 to 100%. They discussed their reasoning with others closest to them and subsequently shared their reasoning with the rest of the class, the teacher, and the researcher. This discussion was moderated by the researcher. Finally, students in the co-design team voted on the percentage of maximum heart rate to use as a threshold when designing their workouts. The researcher guided the voting process by establishing the thresholds that would be included in the vote based on where on the continuum the largest proportions of students were standing. The percentage with the majority vote was used. The same process was used to set the maximum and minimum interval times for work and rest based on students’ understanding of the relationship between heart rate and interval length. During the discussion on interval length, the researcher ensured that it was clear to students that they were able to use any interval length within the minimum and maximum constraints for designing the workouts and that these values were to be used as a guide. Field notes were collected by the researcher to document the discussions and decisions for each parameter. The length of the HIIT workout was predetermined by teachers and researchers based on student ability and time constraints with the intention of using the HIIT workouts in HPE lessons the following term. In school one, 10-minute workouts were created using intervals within the determined constraints. In schools two and three, researchers and teachers decided to have students design a 5-minute workout that would be repeated twice. This decision was based on teacher feedback from school one where teachers felt that students were too rushed during the design and would benefit from more time to focus on the interplay between heart rate and interval length for each of their chosen exercises.

#### HIIT workout creation

Small groups of three to five students created the HIIT workouts during the remaining three lessons using: (1) the criteria developed during the problem identification lesson; (2) the parameters established for the workouts (interval length, heart rate intensity); (3) a booklet of example exercises; and (4) relevant resources identified through the internet. The student groups started by discussing potential group names and themes for their workouts. Afterwards, they proceeded to research and list exercises related to the theme and modifications of the exercises to make them suitable for all levels of ability. They were encouraged to use their heart rate monitors to trial their exercises to ensure the interval length was appropriate and at the desired intensity.

After finalising the first iteration of workouts, the workouts were trialed by the class. Teachers and researchers aided the student groups in leading their workouts with varying levels of involvement based on teacher discretion. Heart rate information was collected, and peer feedback was collected using the criteria and a comment section. In a following lesson, student groups were provided with the feedback and heart rate data. Time was allotted for reviewing and discussing the feedback. Student groups then made any changes they thought would be useful for their final HIIT workout based on feedback and their own experience leading the workout and documented their reasoning. Data collected during the HIIT workout design included the draft HIIT workout, the peer feedback, the heart rate data, the final HIIT workout, and the reasoning for any modifications made by each group.

### Student ownership

Students were informed of their right to equal contribution on par with the researchers and teacher for all activities, such as the criteria creation, the selection of HIIT parameters, and the design of the HIIT workouts. Smaller student groups (3–5 students) were formed to design the HIIT workouts with input from teachers and students. The insight of the teachers into the class dynamics was a valued contribution to the co-design team and enabled them to make an informed decision on how groups should be created. In school one, students were able to vote on how to form the student groups (i.e., by students or teacher) and chose to have the teacher make the decision. In schools two and three, group formation was based on teacher discretion, with students in one class forming their own student groups and students in the second class being organised in student groups by the teacher. Each student group collectively decided their name, workout theme, and exercises to reflect their shared interests.

### Evaluation of feasibility and impact of the Co-Design process

Qualitative data were collected to evaluate the feasibility and impact of the co-design process. Student data included: (1) discussions between each group and a researcher about the co-design process based on a semi-structured guide (all schools) and (2) individual written surveys (School One and School Three). A semi-structured interview was completed with each teacher once the lessons were completed to understand the implementation and integration of the co-design process within the HPE lessons (all schools). This was led by two researchers including the researcher involved in the co-design process, and was audio recorded for subsequent analysis. The teacher interviews were between 20 and 25 min in length, while discussions with student groups lasted approximately 10–15 min each. The survey and discussion guides focused on understanding the implementation and effects of the lessons and are provided in Additional File 1. *Making a HIIT* was completed at each school consecutively and feedback from students and teachers from each school was incorporated into the subsequent school’s co-design process.

### Data analysis

To describe the results of the co-design process (Aim 1), data were collected during each lesson. These data included: (1) the facilitators and barriers to exercise from individual students; (2) the original and modified criteria created by each class; (3) the evaluations of each trialed HIIT workout during the upskilling lesson using the class criteria, which were descriptively analysed; (4) the established HIIT parameters from each class; (5) the heart rate data and evaluations of each pilot HIIT workout, which were descriptively analysed; (6) the themes, time in work, and types of exercises used in each HIIT workout, which were tallied to understand variation between workouts.

To evaluate the feasibility and impact of co-designing HIIT workouts with students and teachers as part of the HPE curriculum (Aim 2), a thematic analysis was completed using the student discussions, student surveys, and teacher semi-structured interviews [27, 28]. Student written responses and discussion notes were collated shortly after the final lesson and teacher interviews were transcribed verbatim within a week of completion by the first author. This was done to increase familiarity with the data. Any personal or identifiable information was deleted. After familiarisation with the data, two authors (S.L.D. and Y.L.) developed the first iteration of the coding framework based on relevant literature on feasibility studies and program evaluations (deductive) and a subset of data from one co-design team (inductive) with a focus on the explicit (semantic) meaning of the text [28]. This was used to code a second subset of data (S.L.D., Y.L., and D. P.) and the coding framework was adjusted based on new content in the data. The coding framework was applied to the rest of the dataset using NVivo (Version R1) over five iterations (S.L.D., Y.L. and D. P.) and discussed until all authors were satisfied with the codes. S.L.D. organised the codes into larger categories in a hierarchical fashion and drafted a thematic map that was discussed and revised by all the authors. The themes were presented with quotes that exemplified each theme.

## Results

### Co-designing the HIIT Workouts

#### HIIT criteria

Compiling the barriers and facilitators of individual students identified similar themes among the participating classes. These included facilitators such as: enjoyment, socialising, and fitness goals; and barriers that included: lacking motivation, feeling tired, being injured, and having no time. Therefore, the criteria for engaging workouts created by the five co-design teams included several common elements: (1) fun; (2) social; (3) achievable skill level; (4) feeling accomplished at the end; and (5) beneficial. However, the manifestation of these criteria differed slightly. For example, the definition of fun for one class included a statement that the exercises shouldn’t be repetitive, while for another class it was expressed as a desire to do the workout. Further, what type of benefit was sought differed in the criteria between classes from health benefits to benefits focused on developing fitness or skills. Compared to the co-educational and girls’ schools, the boys’ school noted competition or a challenge as a facilitator more often, which was reflected in their created criteria (Additional File 2).

After trialling the criteria with the HIIT workouts in the upskilling lesson, two of the five classes modified their evaluation sheets. Originally, the criteria form used a 5-point Likert scale. However, class A at school one determined it would be better to use a 10-point scale to further understand the variability within the feedback and make comparisons among the workouts. This class also added in an additional criterion, “I would do this HIIT workout again”, to inform if a successful HIIT workout had been created. Class D at school three initially only created 4 criteria. However, after trialling the HIIT workouts, they chose to add in a criterion focused on being able to complete the workout at an appropriate level of difficulty. Additionally, they expanded the criterion regarding the required benefits of the HIIT workout to include supporting their physical activity habits.

#### HIIT Workout assessments

Among schools, there were minimal differences in the assessment of the various types of HIIT participated in or trialled in the upskilling lesson. Overall, students in four of five classes rated the relay HIIT workout as the most fun and social (Additional File 3). However, one class in the girls’ school rated the boxing workout as the most fun. Students in all five classes disagreed that the sprint-based workout was fun. However, the sprint-based workout had the highest proportion of “agree” responses for a sense of accomplishment in both classes in the boys’ school.

#### Defining HIIT parameters

The parameters of the HIIT workouts set by the students are displayed in Table 3. Students who argued for a lower heart rate threshold (e.g., 80% of maximum heart rate) felt that it could be more enjoyable and easier to achieve for a greater number of students. They also stated that those who wished to push themselves to a higher heart rate would still have the opportunity as this was only a minimum threshold. Those that defended a higher threshold (e.g., 90% of maximum heart rate) maintained that it would be most suitable for benefits and would enable students to feel more accomplished.

Students that argued for longer work and rest interval maximum lengths reasoned that it didn’t negate the potential to use shorter intervals if they were preferred and they thought it would be wise to have more freedom for the interval lengths. They also argued that for certain exercises there might not be enough time to increase heart rate or to have an appropriate amount of rest after a hard exercise if the maximum interval length was too short. Those in favour of shorter intervals were predominately worried about becoming bored during certain exercises.

#### HIIT workouts

Overall, the co-design teams created thirty-three HIIT workouts (Table 4). All the workouts followed a HIIT format with work and rest intervals that met the HIIT criteria. The percentage of time in work ranged from 50 to 75%. The themes of the workouts are listed in Table 4. Sport warm-ups and general fitness were prominent themes. All the workouts included aerobic components, and 25 included resistance components. The most common resistance exercises included push-ups, sit-ups, and squats. Thirteen workouts included partner exercises. The workouts at school one included music and equipment such as skipping ropes and soccer balls; however, based on feedback from teachers in school one, the use of music and equipment added additional time to complete the workouts during the intervention in the second phase of *Making a HIIT*. Accordingly, and based on consultation with the teachers in schools two and three, music and equipment were removed from these schools.

**Table 4:** High-intensity interval training workout characteristics

School	Class	Team	Theme	Exercise Included			Time in Work
				Partner	Resistance	Aerobic	
One	A	1	Soccer	x		x	65%
One	A	2	Bedroom Workout		x	x	57%
One	A	3	General Fitness	x	x	x	65%
One	A	4	Core & Cardio	x	x	x	53%
One	A	5	Soccer			x	58%
One	A	6	Things Tom Likes	x		x	67%
One	A	7	Volleyball	x		x	68%
Two	B	1	Swimming/Rugby		x	x	67%
Two	B	2	Yard Workout		x	x	57%
Two	B	3	Muscle Burner		x	x	68%
Two	B	4	Football Warmup		x	x	63%
Two	B	5	Home Workout		x	x	62%
Two	B	6	Contact Sports	x	x	x	72%
Two	C	1	General Themed			x	75%
Two	C	2	At Home/Backyard		x	x	63%
Two	C	3	Rugby Themed	x		x	70%
Two	C	4	Volleyball			x	63%
Two	C	5	Team Workout	x	x	x	62%
Two	C	6	General Themed		x	x	72%
Two	C	7	AFL Themed			x	65%
Two	C	8	Send it!		x	x	50%
Three	D	1	Core & Fitness	x	x	x	67%
Three	D	2	Netball		x	x	67%
Three	D	3	Circuit	x	x	x	70%
Three	D	4	Athletics		x	x	57%
Three	D	5	80s Aerobics		x	x	75%
Three	D	6	Work with Friends	x	x	x	67%
Three	E	1	Random		x	x	70%
Three	E	2	Bedroom Workout	x	x	x	75%
Three	E	3	Core		x	x	65%
Three	E	4	Random		x	x	73%
Three	E	5	Everything	x	x	x	53%
Three	E	6	A challenge		x	x	50%

The co-designed high intensity interval training (HIIT) workouts by school, class, and team along with their theme, exercise styles, and the percentage of time each had in work intervals. An ‘x’ in the columns for partner, resistance or aerobic exercises indicates that the workout included at least one exercise in the category

Based on feedback from the co-design team and heart rate data collected during the first pilot, modifications were made to the original HIIT workouts during the second iteration. Most groups changed their interval lengths to add or reduce rest; make it easier to lead /follow if intervals were on the minute. In school one, four of seven groups decided to change the order of their exercises to maintain a higher heart rate as they noticed different exercises produced a different heart rate response. In school two, no groups discussed changing the order of their exercises. Instead, to maintain intensity, three groups discussed adding a goal to the workout interval (e.g., a push-up every couple of seconds for the interval) to encourage their peers to maintain their effort throughout the interval. In school three, five groups changed some of the exercises in their workouts due to difficulty and to include more variety and partner activities to improve the workout’s ability to satisfy the class criteria.

### Feasibility and impact of Co-Designing HIIT in the curriculum

#### Feasibility

The thematic analysis guided the development of three themes and twelve codes and subcodes for the evaluation of lesson feasibility within the curriculum. Feasibility was deductively divided into three main areas based on literature from feasibility studies: (1) acceptability; (2) implementation; and (3) integration of the co-design process within the HPE lessons. The themes and codes are listed with example quotes in Table 5. Of the twelve codes, four were determined deductively based on the areas of focus for feasibility studies by *Bowen et al.* [29] and the other nine were determined inductively based on data.

**Table 5:** Themes and codes on the feasibility and impact of co-designing high-intensity interval training workouts in the curriculum

Theme	Participant Quotes
Code	
**FEASIBILITY**	
**Acceptability**	
Appropriateness	So based on time, it was everything that they could have had, and I think the testament to the whole thing is probably the actual results that we got. (School two, teacher) It all seemed logical and there wasn’t there wasn’t a point where the kids were confused about what to do. Your instructions are very clear. The scaffolded sheet with the example was really good. They always need that gradual release of responsibility and an example. (School three, teacher)
Satisfaction	
Autonomy and choice	The autonomy that they got from designing their own episodes or sessions made them more engaged because they’re not being told what to do. They get to actually have some choice. (School three, teacher) Instead of being assigned, we get to decide what to do. (School three, student)
Inclusive	We all had ideas individually then [we] could discuss and decide on the most effective way, so [we] found this successful. (School one, student) Everyone designed equally, but maybe let every group come up with their own heart rate (HR) threshold then see what they are able to get and then decide on actual threshold. (School two, student)
Enjoyable and engaging	Theory had less writing and was very interactive; I liked it. (School one, student) Yeah, the heart rate monitors were excellent because I said to [the researcher involved in the co-design], because I taught most of them last year, there’s quite a few in that class who don’t like PE and running. It’s so good to see them sprinting across the court because they’re looking at their heart rate. (School three, teacher)
Working with peers	More group work and collaboration time was fun and encourages you to engage more and exposes you to more ideas than you may think of individually. (School one, student) It was beneficial cause I couldn’t have thought about the answer by myself. (School three, student)
**Implementation**	
Processes	I remember [the researcher] and I had a conversation, and [the researcher asked if I thought] we should put them in groups or let them choose their groups. And I think that was a big difference. I think if we’d placed them in groups, they may not have been receptive because not everyone friends and it may not have been as successful. (School three, teacher) Delivery by students was too difficult. Maybe [in future] they demonstrate, but [the workout] is led by the teacher. (School one, teacher)
Facilitators of Implementation	The initial barriers and facilitators activity with the sticky notes was just a nice different way of doing the session. You could have just literally got them to write it down and it would have been a lot different but getting up and grouping it was engaging and I think that was kind of a hook to begin with, like this is how it’s going to go. If it was you just talking, it would have been very different, so that was good. (School three, teacher) My favourite things about this was the heart rate monitors. (School three, student)
Challenges	Sometimes confusing because most have different ideas and want different things. We had to agree so picked something that we all wanted to do. (School three, student) What was harder for us I guess was we had the one with where we jammed a few things in and the main one of them evaluating each other HIITs, we had like 15 min less because of the house choir day. It would have been better if they were able to do a few more of each other’s, but I think they did embrace it, definitely. (School two, teacher)
**Integration**	
Perceived fit in the curriculum	We’re across the middle school from Years 7 to 9 so there’s obviously so many different descriptors we hit. There’s a couple of those that have health benefits. (School two, teacher) I think it was a bit hard this term, like our girls were doing softball and then this, but they’ve done a health and fitness unit last year for year seven, so it’s really complementing that because we’ve done some different workouts and different things, whether they remember them or not. So, it really complements that, but it’s really helpful for them to use in lessons just for fitness because there is a lack of fitness. (School three, teacher)
Perceived sustainability	I would use some or all of [the workouts] because they got my heart rate up and were beneficial. (School one, student) [I would continue to use] the barriers and facilitators, figuring out why young people don’t like to exercise or what motivates them to, because if we can get past that, then that’s a good starting point. (School three, teacher)
Future suggestions	Maybe not 10 min, maybe 5 min and they repeat. Students struggled to find that many exercises for a theme and forget that they can repeat. (School one, teacher) Maybe more encouragement and use music during our HIIT workout to hype up students. (School three, student)
**IMPACT**	
**Educative outcomes**	
Health benefits of HIIT	We learned what a HIIT workout was and the health benefits of HIIT. We learned about the factors that influence fitness and the types of fitness. (School one, student) I learnt that exercise is important for your health and don’t be afraid to challenge yourself. (School three, student)
HIIT specific knowledge	I learnt how to reach a high intensity in a short amount of time; I learnt what HIIT is and what it is about. (School one, student) I mean before [Steph] did that theory lesson, they had no idea what high intensity was. Where you did that activity - stand here, here or here with the intensity – and that’s their curriculum correlation. They’ve never known that. (School two, teacher)
Student barriers and facilitators to exercise	I learnt that you can push yourself further than you thought. You actually feel good after workout. (School three, student) My favourite part was the sticky notes. Seeing people didn’t work out for the same reasons. (School three, student)
**Perceived changes to lessons**	
	An activity like this or this kind of session allows them to have that autonomy; participation; they can learn from each other. It’s just different to what we’re typically used. (School three, teacher) [We] created our own instead of mindlessly going with what the teacher says. (School three, student)
**Personal and social capabilities**	
	[I was able to] expand my social skills when co-creating HIIT exercises. (School one, student) They learn a lot of like management skills and like how to cooperate with each other and that not every person’s opinion is going to be used. (School three, teacher)

Themes and codes generated from semi-structured interviews with teachers, discussion groups with students, and student surveys, related to the co-design of HIIT workouts within the HPE curriculum. This table only includes two illustrative participant quotes per code, thereby providing a summary of the full dataset. School and participant information have been included in round brackets. When necessary, the subject of a sentence has been added in square brackets or tense corrected

##### Acceptability

Acceptability was defined as how the intended recipients reacted to the co-design process [29]. All five co-design teams (students and teachers) discussed their satisfaction with *Making a HIIT*. Overall, students enjoyed the opportunity to work in groups and found the nature of the co-design process provided them more freedom and ownership during HPE compared to normal lessons. They also communicated that the lessons enabled an inclusive environment where their opinions were valued. Students expressed that they found the lessons engaging. Similarly, teachers noted a high level of engagement from students, especially when using the heart rate monitors and during group activities where they had more freedom. They also expressed that the lesson content and classroom organisation were appropriate for the students and noted that students were almost always on-task and responsive to the tasks that they needed to complete.

##### Implementation

Implementation was defined as the extent to which the co-design lessons were implemented as planned within HPE [29]. Certain aspects of the lessons were noted by teachers to facilitate the implementation of the co-design process, such as: (1) the sticky notes and active group work that were used to understand students’ barriers and facilitators to exercise; (2) the heart rate monitors for encouraging high intensity while performing the workouts; and (3) the booklet of exercises, which allowed students to have a base for creating their workouts. Students unanimously expressed that they enjoyed seeing their heart rate projected in the gymnasium. They communicated that the researcher delivering the lessons was friendly and they liked working with her, which teachers attributed towards the commitment that students demonstrated towards the co-design process. Both the students and teachers noted similar challenges within the lessons stemming from disagreements within the groups and learning to collaborate and discuss differences of opinion. Teachers also expressed challenges related to time constraints. These were due to both external demands that resulted in less time dedicated to the co-design process than expected and due to the amount of material in some lesson, especially with attempting to pilot all the HIIT workouts. The teachers’ suggestions regarding how to address perceived challenges are listed below under *Integration*. Finally, teachers discussed the general process of the implementation in their specific class. Pending the class dynamics, teachers either gave the students more freedom and responsibility when choosing their groups and piloting the workouts or provided more guidance. They explained that making some decisions without the entire co-design team (e.g., without student input) enabled smoother implementation due to classroom dynamics and time constraints in the lessons.

##### Integration

Integration was defined as the extent the co-design process was integrated within the existing curricula and school units [29]. Overall, teachers expressed that the lessons aligned with the Australian HPE curriculum for Years 7 and 8. However, they also noted that the lessons could fit with the curriculum of more senior years where students are tasked with designing fitness programs. All five teachers stated that there were parts of the lessons that they intended to use again, including the sticky notes and heart rate monitors, with one school investigating the use of the Polar GoFit software for other units. Teachers also provided recommendations for the co-design process in future schools, such as shortening the length of the co-designed workout from 10 min to 5 min and adding additional scaffolding to the first iteration of the HIIT workout design due to time constraints. Some students stated that they intended to continue using the workouts that they created, while others said they preferred team sports or workouts based on repetitions instead of timed intervals and would likely not use the workouts moving forward. Students from schools two and three, who did not have the opportunity to use music for their HIIT workouts, recommended its use to increase motivation.

### Impact

Impact was defined as the significance, usefulness, or benefit of the co-design process [30]. Three themes (*Perceived changes to lessons*; *educative outcomes*; and *personal and social capabilities*) and three codes were identified in the data related to impact (Table 5). Students and teachers perceived changes in the lessons compared to normal HPE lessons with additional interaction and active participation involved in the co-design process. The lessons also supported educative outcomes related to the HPE curriculum. Students expressed that they had gained knowledge related to HIIT and the health benefits associated with both HIIT and exercise in general. Further, students expressed that they learned about their barriers and facilitators toward exercise and how to motivate themselves to work harder than they would normally. Lastly, teachers and students both acknowledged that students improved their social and personal capabilities in line with the curriculum through improved confidence, compromise, collaboration, and team management skills.

## Discussion

The co-design process within *Making a HIIT* was documented in detail in this paper to provide a comprehensive and transparent understanding of how the lessons integrated within the HPE curriculum and its potential to be used within schools in a meaningful manner. The process led to the successful creation of 33 HIIT workouts within the three participating schools, demonstrating that students aged 12–14 years are capable of understanding the interaction between intensity and interval duration and applying it to design a HIIT workout targeted at satisfying agreed upon HIIT parameters and evaluation criteria. The co-designed workouts contained a variety of exercises and a range of work-to-rest intervals. They included greater variation than standardised running protocols, which are the most commonly used in this setting [8]. The increased variety and student ownership could have a positive influence on students’ engagement with the workouts moving forward [24].

Overall, in this study, co-designing HIIT workouts with students and teachers was perceived to be feasible within the HPE curriculum. Previous literature involving children and varying levels of co-design participation corroborates the feasibility of this type of work. For example, school-aged children have previously been involved in the design of healthy dairy products [31], the design of school buildings [32], new technology [33], and the curriculum [34]. Further, the thematic analysis identified that both students and teachers were largely satisfied with the *Making a HIIT* co-design process. Similarly, a recent study that incorporated HIIT in two schools in New Zealand included teacher input on curriculum connections for HIIT and was well-received by teachers and appeared to enhance the buy-in of the HIIT intervention [35]. In *Making a HIIT*, teachers occasionally determined that certain decisions needed to be made without student input in the interest of time. For example, student groups at school one was able to use equipment (e.g., skipping ropes, balls) and music during their HIIT workouts and students noted that they enjoyed these aspects of the workouts. However, based on teacher feedback from the implementation of the intervention in this school, these options were removed for future schools. Similarly, there were differences between student and teacher opinion on the formation of the small groups that were used in the co-design process. Students almost unanimously preferred to choose their own groups; however, on two occasions teachers determined that due to student dynamics it would be necessary for them to have additional influence on the group creation. The class dynamics and lesson time constraints also influenced how much freedom teachers were willing to provide students for piloting the HIIT workouts. Even though these areas that afforded student autonomy were removed based on teacher discretion and constrained the co-design task to narrower boundaries, students still noted that the co-design process included more active participation, freedom, and choice compared to their standard HPE lessons. Future studies considering similar implementation within lessons need to consider the adaptability of the lessons to meet different teacher preferences and class dynamics. The expertise of the teachers as a member of the co-design team was vital throughout the process due to their relationship with the students and should not be overlooked. Their knowledge of the class dynamics, lesson structure, and school structure assisted in the design of workouts with the potential for successful implementation and sustainability in their specific school.

Students and teachers identified that the lessons provided the intended educative outcomes related to the HPE curriculum, but additionally provided outcomes related to the development of students’ personal and social capabilities, which is part of the general capabilities targeted in the curriculum [15]. The personal and social capabilities are characterised by development in both self and social management and include sub-elements such as confidence and adaptability; appreciation of diverse perspectives; working collaboratively; resolving conflict; and developing leadership skills. These additional outcomes, which are afforded by the increased student ownership and autonomy in co-design, expand the potential opportunities for co-design in schools beyond HPE by contributing to broader curriculum aims. Another outcome of the co-design process was that students expressed that they had a greater appreciation of how to create enjoyable and motivational workouts that they would continue to use beyond the scope of the lessons. Further, they shared that they gained an understanding that not all students had the same point of view on what constituted an enjoyable workout. Together, it can be argued that these outcomes contributed to improved physical literacy of the participating students. Physical literacy includes four elements: (1) the affective domain (e.g., motivation and confidence); (2) the physical domain (e.g., fundamental motor skills); (3) the cognitive domain (e.g., knowledge and understanding); and (4) the behavioural domain (e.g., lifelong engagement in physical activities), which were all identifiable in the co-design process [36]. Improving students’ physical literacy is noted by Sports Australia in the Australian Physical Literacy Framework to be important for positive lifelong physical activity behaviours, which is an encouraging outcome beyond the successful co-design of HIIT workouts [36].

### Strengths and limitations

This is the first study to embed the design of HIIT workouts within the curriculum in collaboration with teachers and students in an attempt to mitigate some of the typical challenges experienced in school-based research, such as the overburdening of teachers and curricular demands [9]. The co-design process has the potential to increase the sustainability of HIIT workouts through the creation of more engaging workouts for teachers and students [24]. However, due to the heavy involvement of a researcher, wider dissemination of this work would require modifications. This could include developing professional training on HIIT and the process of co-design for teachers, and resources that teachers could use to lead the design of HIIT workouts with students. While integrating *Making a HIIT* within the curriculum afforded important outcomes, several aspects of student ownership could not be completed as initially planned by the research team due to time and space restrictions (e.g., students adding music to workouts, using various pieces of equipment, or choosing the location for the HIIT workouts). These elements could be incorporated in future interventions depending on time availability and the context of the co-design as it is adapted for varying schools. However, even without these elements, students still noted opportunities for active participation, choice, freedom, and sharing of their ideas. Due to the amount of time allotted to the process and the size of participating classes, the feedback captured from the co-design team could not always be as detailed as desired. However, having data from both students and teachers provided a strong overarching evaluation of the process. Further, as the lead author was involved in the co-design process, we must acknowledge that we have subjective biases within the interpretation of these results. However, this involvement with the co-design process and interviews also enabled a more nuanced commentary on the findings. Further, the inclusion of multiple authors in the qualitative data analysis ensured that the views were discussed and agreed upon by all participating authors. While this study was conducted in Queensland, Australia using the relevant HPE curriculum content descriptions, most HPE curricula include similar elements and standards for general fitness units. Therefore, with appropriate modifications, this type of co-design could be integrated in HPE curricula elsewhere in Australia or globally.

### Conclusions and future directions

Co-designing HIIT workouts with students and teachers resulted in the successful creation of HIIT workouts that were aligned with set HIIT parameters and the developed criteria for engaging workouts. The process was found to be feasible within HPE lessons and contributed positively to students’ educative outcomes. It also provided students with additional autonomy and choice compared to normal HPE lessons. Future studies focused on HIIT interventions in schools should consider the use of co-design or a similar process to understand the integration and maintenance of HIIT programming within the school context. As each school is unique, recent recommendations suggest using a context-specific approach when implementing and scaling interventions, where certain intervention components are essential, while others are modifiable [37]. In the context of *Making a HIIT*, an essential component could be providing teachers with the lesson plans that could be integrated either in full or part within the curriculum for HIIT workout design, while affording students additional autonomy and choice in their lessons. The next phase of *Making a HIIT* will examine the effect of co-design on students’ motivation, enjoyment, and self-efficacy towards the HIIT workouts when used in an intervention.

### Supplementary Information

** Additional file 1.** Discussion and interview guides for evaluating the co-design process. The discussion guide for students, the semi-structured interview guide for teachers and the student written survey questions used to evaluate the co-design process.

** Additional file 2.** High-intensity interval training criteria for each co-design team (class). The criteria created by each co-design team based on facilitators and barriers that were identified during the first co-design lesson and the modifications made to the criteria after trialling them with pre-made high-intensity interval training workouts.

** Additional file 3.** Student Evaluations of HIIT Workouts from the HIIT Upskilling Lesson. The evaluations of the pre-made HIIT workouts in each of the five co-design teams. In school two, the dancing and boxing HIIT workouts were not completed and in school three, the dancing HIIT workout was not completed. Class D originally only included four criteria. The graphs indicate how many students agreed, disagreed, or were neutral towards each criterion as a percentage of the class. HIIT = high-intensity interval training.

## Data Availability

The data analysed during this study are available from the corresponding author on reasonable request.

## Abbreviations

HIIT: high-intensity interval training
HPE: Health and Physical Education
IAP2: International Association for Public Participation
