# Supplementary material for: Making a HIIT: co-design of high-intensity interval training workouts with students & teachers within the curriculum
Source: BMC Public Health. 2023 Sep 15;23:1795. doi: 10.1186/s12889-023-16613-8 (PMC10503108; doi:10.1186/s12889-023-16613-8)
Supplement: Supplementary file 1 — Additional file 1. Discussion and interview guides for evaluating the co-design process. The discussion guide for students, the semi-structured interview guide for teachers and the student written survey questions used to evaluate the co-design process. [file 12889_2023_16613_MOESM1_ESM.docx]

**Additional File 1.** Discussion and interview guides for evaluating the co-design process.

**Teacher Interview Guide**

1. Overall, how did you feel that the lessons went?
2. How, if at all, has this process complemented the work you do as part of the curriculum?
3. What aspects of the co-design process, if any, would you use again? Why?
4. What aspects of the process, if any, do you think were best received by the students? Why?
5. What aspects of the process, if any, do you think could be improved?
6. Is there anything you would add to or modify within the lessons?

**Student Group Discussion Guide**

1. Over the last three weeks, we have been working together to design HIIT workouts. Have you noticed any differences in the lessons you have done with [researcher name inserted] compared to your normal lessons?
2. How did you find working together to co-design the HIIT workouts and why?

(With researcher, with teacher, with other peers)

1. How/Why did you choose the 1) theme/ 2) exercises/ 3) intervals for your workout?
2. What did you change in your workout after you tried it and why?

**Student Individual Written Survey**

1. What are two or three things that you learnt throughout the 6 lessons?
2. What was your favourite and least favourite part about the 6 lessons?
3. What, if anything, was beneficial about co-creating HIIT workouts together? What, if anything, was not beneficial?
4. If we were to do this again, would you change anything or want us to change anything?
5. Which of the workouts that we co-created, if any, would you use again? Why?
