# Supplementary material for: Making a HIIT: co-design of high-intensity interval training workouts with students & teachers within the curriculum
Source: BMC Public Health. 2023 Sep 15;23:1795. doi: 10.1186/s12889-023-16613-8 (PMC10503108; doi:10.1186/s12889-023-16613-8)
Supplement: Supplementary file 2 — Additional file 2. High-intensity interval training criteria for each co-design team (class). The criteria created by each co-design team based on facilitators and barriers that were identified during the first co-design lesson and the modifications made to the criteria after trialling them with pre-made high-intensity interval training workouts. [file 12889_2023_16613_MOESM2_ESM.docx]

**Additional File 2.** High-intensity interval training criteria for each co-design team (class).

**School One Class A**

1. I found the exercises in this workout fun
2. During this workout, I felt that I had choices to appropriately challenge myself and the workout was inclusive of my skill level
3. I felt like doing this with my friends and classmates made it more enjoyable
4. I had a sense of accomplishment at the end of this workout
5. I felt that I got my heart rate high enough to enable greater health benefits during this HIIT workout
6. I would do this HIIT workout again*

**School Two Class B**

1. I found the exercises in this workout fun and not too repetitive
2. During this workout, I felt that I had choices to appropriately challenge myself and the workout was not too difficult for me
3. I felt like doing this with my friends and classmates made it more enjoyable
4. I felt like I had a goal to work towards
5. I felt that this HIIT workout could help me improve my fitness and/or my skill levels

**School Two Class C**

1. I found the exercises in this workout fun, and it was something that I wanted to do
2. During this workout, I felt that I had choices to appropriately challenge myself and the workout was inclusive of my skill level
3. I felt like doing this with my friends and classmates made it more enjoyable
4. I had a sense of accomplishment at the end of this workout
5. I felt that I got my heart rate high enough to enable greater health benefits and improve my fitness during this HIIT workout

**School Three Class D**

1. I found this workout fun
2. I felt like doing this with my friends and classmates made it more enjoyable
3. I had a sense of accomplishment and success at the end of this workout
4. I felt like this workout was the appropriate level of difficulty for me*
5. I felt that participating in this workout supports my health and my physical activity habits*

**School Three Class E**

1. I found the exercises in this workout fun
2. I did a good job of completing this workout at an appropriate level
3. I felt like doing this with my friends and classmates made it more enjoyable
4. I had a sense of accomplishment and success at the end of this workout
5. I felt that this HIIT workout helped support my fitness and/or health goals

*Underlined components were added in the second iteration of the criteria.
