# Supplementary figures and images for: Making a HIIT: co-design of high-intensity interval training workouts with students & teachers within the curriculum
Source: BMC Public Health. 2023 Sep 15;23:1795. doi: 10.1186/s12889-023-16613-8 (PMC10503108; doi:10.1186/s12889-023-16613-8)

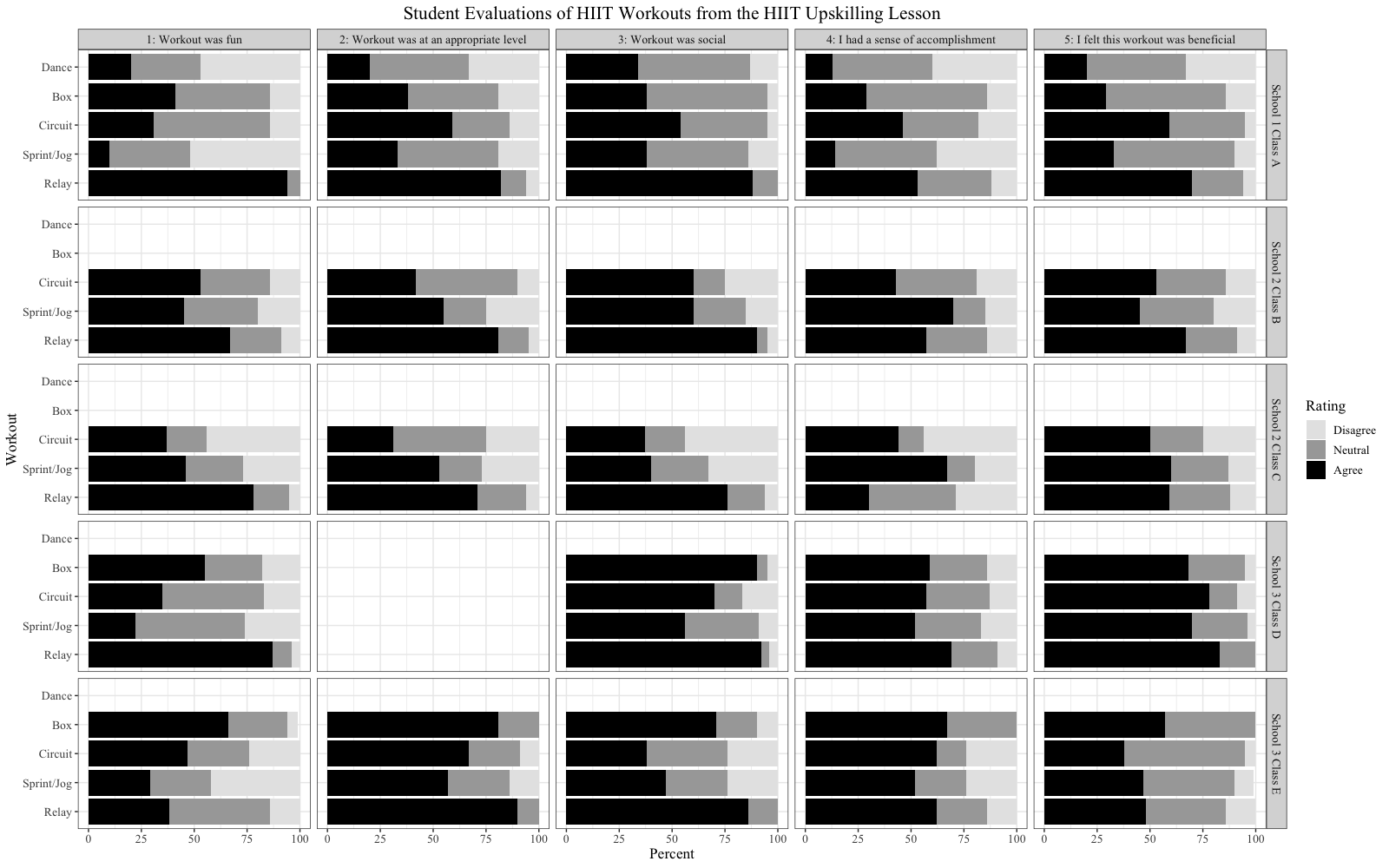

Supplement: Supplementary file 3 — Additional file 3. Student Evaluations of HIIT Workouts from the HIIT Upskilling Lesson. The evaluations of the pre-made HIIT workouts in each of the five co-design teams. In school two, the dancing and boxing HIIT workouts were not completed and in school three, the dancing HIIT workout was not completed. Class D originally only included four criteria. The graphs indicate how many students agreed, disagreed, or were neutral towards each criterion as a percentage of the class. HIIT = high-intensity interval training. [file 12889_2023_16613_MOESM3_ESM.png]
